# Premature drug reduction after subthalamic nucleus deep brain stimulation leading to worse depression in patients with Parkinson's disease

**DOI:** 10.3389/fneur.2023.1270746

**Published:** 2023-10-19

**Authors:** Yu Diao, Tianqi Hu, Hutao Xie, Houyou Fan, Fangang Meng, Anchao Yang, Yutong Bai, Jianguo Zhang

**Affiliations:** ^1^Department of Neurosurgery, Beijing Tiantan Hospital, Capital Medical University, Beijing, China; ^2^Department of Functional Neurosurgery, Beijing Neurosurgical Institute, Capital Medical University, Beijing, China

**Keywords:** Parkinson's disease, short-term effects, follow-up, deep brain stimulation, propensity score matching, depression, non-motor symptoms, medicine reduction

## Abstract

**Background:**

Reduction of medication in Parkinson's disease (PD) following subthalamic nucleus deep brain stimulation (STN-DBS) has been recognized, but the optimal timing for medication adjustments remains unclear, posing challenges in postoperative patient management.

**Objective:**

This study aimed to provide evidence for the timing of medication reduction post-DBS using propensity score matching (PSM).

**Methods:**

In this study, initial programming and observation sessions were conducted over 1 week for patients 4–6 weeks postoperatively. Patients were subsequently categorized into medication reduction or non-reduction groups based on their dyskinesia evaluation using the 4.2-item score from the MDS-UPDRS-IV. PSM was employed to maintain baseline comparability. Short-term motor and neuropsychiatric symptom assessments for both groups were conducted 3–6 months postoperatively.

**Results:**

A total of 123 PD patients were included. Baseline balance in motor and non-motor scores was achieved between the two groups based on PSM. Short-term efficacy revealed a significant reduction in depression scores within the non-reduction group compared to baseline (*P* < 0.001) and a significant reduction compared to the reduction group (*P* = 0.037). No significant differences were observed in UPDRS-III and HAMA scores between the two groups. Within-group analysis showed improvements in motor symptoms, depression, anxiety, and subdomains in the non-reduction group, while the reduction group exhibited improvements only in motor symptoms.

**Conclusion:**

This study provides evidence for the timing of medication reduction following DBS. Our findings suggest that early maintenance of medication stability is more favorable for improving neuropsychiatric symptoms.

## Introduction

Deep brain stimulation (DBS) of the subthalamic nucleus (STN) is the first-line surgical treatment for Parkinson's disease (PD), it can effectively improve Parkinson's disease patients' motor and non-motor symptoms (1, 2), reduce motor fluctuations, reduce dopaminergic drug use and adverse drug reactions, and improve patients' quality of life (3–5). In recent years, an increasing number of studies have discovered that non-motor symptoms, particularly emotional aspects such as anxiety and depression, have a significant impact on the quality of life of Parkinson's disease patients (6).

The success of DBS is critically dependent on delivering the appropriate dose of stimulation at the best location within the target region (7). DBS programming is the process of choosing the appropriate electrical stimulation dose at the individual electrode placement and anatomy to achieve the greatest clinical benefit (8). DBS programming is a process that relies on clinical observation and repeated empirical attempts by programming doctors (9). A large number of parameter combinations, including frequency, pulse width, and stimulation voltage selection, as well as contact selection, are usually insufficient to test the best curative effect in a clinical routine treatment (10, 11). If the patient experiences drug side effects such as dyskinesias, apart from the stimulation parameters, the drug may also be adjusted (12). This will result in a greater number of test parameters being combined. Furthermore, while real-time observation and adjustment of stimulation parameters and contacts can improve motor symptoms, most non-motor symptoms cannot be effectively adjusted through real-time observation (13, 14). As a result, the curative effect of programming is highly dependent on the programming doctor's experience and the number of times the patient has gone through trial and error. This has resulted in patients' uncontrollable postoperative clinical benefit, with individual differences in clinical benefit.

In addition to improving the clinical symptoms of patients, STN-DBS has the effect of reducing dopamine drug use (15). Existing research indicates that after DBS, a high dose of LEDD reduces quality of life, and that there is a negative correlation between the two (16). As a result, postoperative drug adjustment, especially the reduction of drug, is critical. Compared with drug therapy alone, the adjustment of drug dosage and type after STN-DBS can better improve the patients' motor and non-motor symptoms (4, 17, 18). For example, after surgery, appropriate medication reductions improved the patient's depressive symptoms and reduced motor fluctuations (19, 20). Medication is reduced based on response to stimulation according to existing drug reduction strategies. If there is a clear response to stim, even a partial response, medication can be gradually reduced. If there is not a response to stimulation because the stimulation parameters are still being determined, medication should not be reduced (21). However, the current drug reduction strategy only relies on clinical response and doctor experience, which has some limitations. If the patient's first-time programming effect is satisfactory, it is an unresolved issue whether the drug dosage should be reduced immediately or maintained in the early stage. If the dosage, type, or timing of the adjustment of the drug is improper, it may cause dopamine dysregulation syndrome (DDS), mental symptoms, depression, mania, etc. (22–24). Improper adjustment of dopamine agonists within 3 months after DBS can lead to the development of manic symptoms or impairment of impulse control (25). In addition, reducing dopamine agonist medications and levodopa immediately after surgery can lead to depression and anxiety (26). As a result, the current DBS postoperative drug adjustment is also based on trial-and-error results of the programmed parameters and the experience of the programmed doctor, and the current postoperative drug adjustment lacks clinical evidence support. When the curative effect of the patient's first programming is acceptable or dyskinesias occur, the programming doctor usually chooses to reduce the drug dosage or stimulation parameters, however, there is no evidence to suggest which method is superior.

In the present study, we included 123 patients treated with STN-DBS and compared early drug reduction (drug reduction at the initial programming after surgery) and late drug reduction (reduction of drug after 3–6 months after surgery) differences in motor and mental symptoms, with the goal of providing clinical evidence support for drug adjustment after DBS and reducing the number of programming trial and error.

## Methods

### Study design and ethical approval

This study was a single center retrospective study. Baseline visit was conducted between August 2018 and February 2021 in Beijing Tiantan Hospital. The programming parameter of the first time was high frequency stimulation, as used by most centers.

The patients in this study were all treated at Tiantan Hospital, and the study was conducted in accordance with the Declaration of Helsinki and authorized by Tiantan Hospital Ethics Committees. Our study followed the STROBE guidelines, and the selection of clinical problems followed the PICOS principle.

### Patients

The PD diagnosis was based on UK Brain Bank criteria (27). Patients were screened for bilateral STN-DBS according to the International PD and Movement Disorders Society and National Neurological Society guidelines (28). Levodopa challenge tests were considered satisfactory if there was >30% Movement Disorders Society-Unified Parkinson's Disease Rating Scale (MDS-UPDRS)-III improvement, and the patients were treated with DBS.

Patient medication adjustments were jointly decided by neurologists and programming doctors. Initial programming for patients commenced at 4–6 weeks post-surgery (T1) and spanned a duration of 1 week, involving 2–3 programming sessions. During this period, medication adjustments were based on the patients' MDS-UPDRS Part IV item 4.2 scores. Patients with scores ≥2 points after 2–3 programming sessions underwent medication reduction, while those scoring 0–1 points had their medication maintained at a stable level. Short-term follow-up at 3–6 months post-surgery (T2) involved maintaining medication dosages unchanged from T1. Patients were categorized into reduced and non-reduced groups based on whether medication adjustments were made at T1. We excluded patients with clinically relevant severe neuropsychiatric disorders and if neuropsychological impairments were found in the preoperative multi-disciplinary assessments by specialized neuropsychologists and neuropsychiatrists (29). All participants provided written informed consent to take part in the study.

### Clinical assessment

Clinical assessments were conducted at preoperative baseline (within 1 month before surgery) in the ON-medication state (MedON) and OFF-medication state (MedOFF), with at 3–6 months follow-up (medication and stimulation ON/medication OFF and stimulation ON, MedON-StimON/MedOFF-StimON). Due to the best efficacy of DBS achieved within 3–6 months after surgery, 3–6 months was the time point for short-term efficacy analyses of this cohort study at our center (30).

With the following scales:

1. Motor disorder: MDS-UPDRS-III (31) and Hoehn-Yahr Stage were used to investigate motor impairment. MDS-UPDRS-III involved video collections and was scored by two experienced doctors in a single-blind way, back-to-back (32, 33). Higher scores indicated higher impairment in all scales.
2. The therapeutic medical regimen was recorded to calculate the levodopa equivalent daily dose (LEDD) according to the method of Tomlinson et al. (34).
3. The Hamilton Rating Scale for Anxiety/Depression (anxiety/depression, HAM-A/-D) were used for anxiety and depression (35). The 24 items of HAMD were grouped into the following seven factors: (1) anxiety/somatization (six items: psychic anxiety, somatic anxiety, gastrointestinal symptoms, hypochondriasis, insight, and general symptoms); (2) weight loss (one item); (3) cognitive disturbances (six items: self-guilt, suicide, agitation, depersonalization and derealization, paranoid, and obsessive compulsive symptom); (4) circadian fluctuations (one item); (5) retardation symptoms (four items: depression, work and interests, retardation, and sexual symptoms); (6) sleep disturbances (three items: difficulty falling asleep, superficial sleep and early awakening); (7) hopelessness symptoms (three items: helplessness, hopelessness, and worthlessness) (36). The 14 items of HAMA were grouped into the following two factors: (1) somatic anxiety (seven items: somatic anxiety muscular, somatic anxiety sensory, cardiovascular symptoms, respiratory symptoms, gastro-intestinal symptoms, genito-urinary symptoms, and autonomic symptoms); (2) psychic anxiety (seven items: anxious mood, tension, fears, insomnia, cognitive, depressed mood, and behavior at interview). Higher scores indicated higher impairment in all scales.

## Programming and follow-up

In Tiantan Center, there is a special team responsible for PD patients' evaluation. They did not participate to the programming or surgery procedure. So they were unaware of the programming parameters or whether the drug was reduced. At each follow-up, an appointment was made for the patients to be evaluated first, followed by programming. We considered these results were less bias for acute programming parameter changes. As a result, the evaluation results were guaranteed to reflect the patient's true effects within 3–6 months.

The programming doctor performs trial and error on the patient's programming parameters based on the patient's motor symptoms, non-motor symptoms, dyskinesias, and other side effects. The initial programming parameters primarily employ monopolar stimulation of the STN's dorsal contact, 130 Hz high-frequency stimulation, 60 μs pulse width, and a voltage of approximately 2.0 V. Adjust the combination of contacts and parameters based on the patient's immediate motor response and stimulus-related side effects to the programming when the patient is not taking anti-parkinsonian drugs.

Following 1 week of programming and observation, patients conclude their initial programming phase. Subsequently, they return to the hospital for short-term follow-up 3–6 months postoperatively. Throughout the T1-T2 timeframe, the patients' programming parameters and medication regimens remain consistent with those at T1. After the conclusion of the short-term follow-up, patients undergo another 1 week programming and medication adjustment session. We define 1-year post-surgery as the milestone for long-term follow-up, at which point we conduct an assessment of the patients' long-term therapeutic efficacy.

### Statistical analysis

The normality of distribution was assessed using the Shapiro-Wilk test. Differences of baseline characteristics and the follow-up between the two groups were analyzed using Mann-Whitney U-tests or unpaired *t*-tests, if parametric tests were applicable. Outcome changes from baseline to follow-up of each group were tested using Wilcoxon signed-rank tests or paired samples *t*-tests, and the results from the two groups were tested with Mann-Whitney U-tests or unpaired *t*-tests. The threshold was *P* < 0.05, unless stated otherwise. All statistical tests were two-tailed. The magnitude of clinical responses was evaluated with relative changes [(mean Test_follow − up_-mean Test_baseline_)/mean Test_baseline_] and Cohen's effect size [(mean Test_baseline_-mean Test_follow − up_)/SD Test_changescores_]. Confidence intervals were calculated for effect sizes based on non-central *t*- distribution (29). Multiple comparisons of the domines of HAMA and HAMD, resulting from the two groups and the use of multiple tests, were corrected using the FDR method. Corrected *P*-values adjusted to the significance threshold of *P* < 0.05 are presented unless otherwise stated.

Furthermore, as our study included data from an observational perspective cohort study of MDS-UPDRS-III, we screened all patients who met the enrollment criteria of this study, and who were enrolled from August 2018 to February 2021, and excluded patients who were lost to follow-up. We used propensity score matching (PSM) to increase causal inference and minimize selection bias. PSM is therefore also called a post-random method. The aim here was to find sub-cohorts of patients in the reducing LEDD group and non-reducing LEDD group with accurately balanced preoperative demographic and clinical characteristics. Variables included for propensity score matching were baseline MDS-UPDRS-III (MedOFF), LEDD, HAMD, HAMA, age at intervention, sex, and duration to reduce confounding factors between categories. Nearest-neighbor matching with a 0.03 caliper without replacement was conducted using a 1:1 ratio (37–39). Subsequently, all analyses of clinical changes from baseline to follow-up were also conducted for the identified matched cohort. Statistical analyses were performed using SPSS statistical software for Windows, version 25.0 (SPSS, Chicago, IL, USA) and R 3.6.1 (R Foundation for Statistical Computing. Analyses were performed using the R statistical software version 3.6.1 (available online at http://www.R-project.org) and propensity score analysis using the “MatchIt” package (40).

## Results

**Figure 1:**
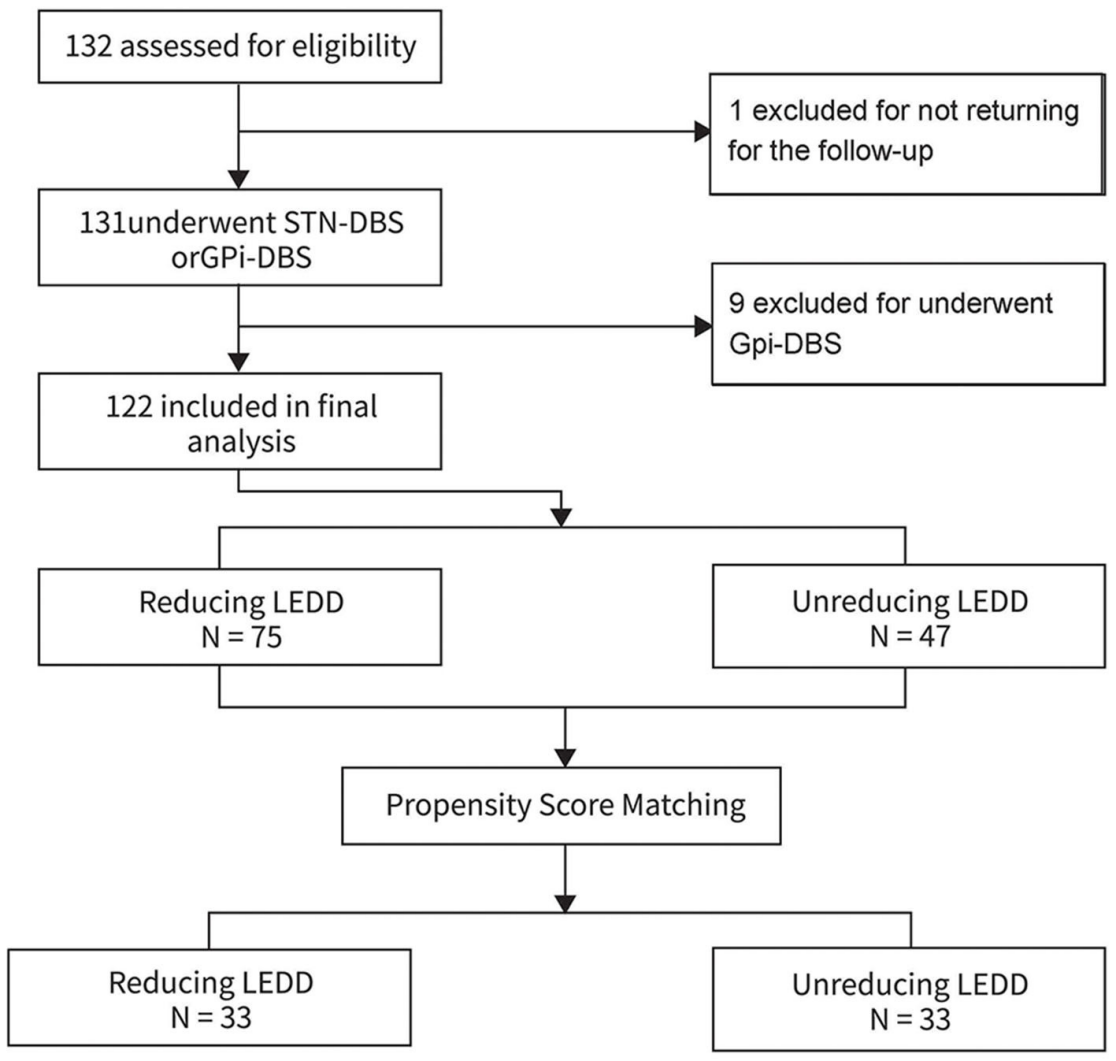
Enrollment.

A total of 123 patients were included, and one patient who was not returning for the 3–6 months follow-up were excluded. Finally, a total of 122 patients (64 males) with PD patients undergoing bilateral STN-DBS were included in the statistical analyses (Figure 1). The patients were divided in two groups involving the reducing LEDD group (*n* = 75) and non-reducing LEDD group (*n* = 47). The 122 patients in the final analysis were aged 61.93 ± 8.41 years with 13.21 ± 4.75 years disease durations. The Hoehn and Yahr was 2.9 ± 0.2. The average MMSE was 26.19 ± 4.04 and the cores of PDQ-39 were 60.83 ± 24.21 for all patients.

### Baseline characteristics

Comparing baseline parameters of the two groups in the original cohort (Table 1), we observed significantly less LEDD in the non-reduced LEDD group (*P* = 0.001). In addition, there was no significant difference in the baseline age, sex, and duration of disease between the two groups.

**Table 1:** Demographic characteristics and outcome parameters at baseline in unmatched and matched cohorts.

	**Original cohort**						***P***	**Matched cohort**						***P***
	**Reducing LEDD**			**Unreducing LEDD**				**Reducing LEDD**			**Unreducing LEDD**			
	***n***	**Mean**	**SD**	***n***	**Mean**	**SD**		***n***	**Mean**	**SD**	***n***	**Mean**	**SD**	
Age	75	61.57	8.62	47	62.5	8.12	0.432	33	62.36	6.29	33	63.70	7.02	0.419
Disease duration	75	12.88	5.06	47	13.74	4.20	0.275	33	14.03	4.07	33	13.15	4.02	0.380
Sex (F/M)	75	(35/40)	46/54%	47	(21/26)	44/56%	0.432	33	15/18	45/55%	33	12/21	36/64%	0.45^a^
MDS-UPDRS-III (med on)	75	23.27	15.82	47	20.62	11.63	0.528	33	23.96	16.15	33	23.01	10.94	0.780
MDS-UPDRS-III (med off)	75	50.29	17.55	47	50.36	22.27	0.985	33	53.54	18.04	33	54.26	24.17	0.892
LEDD	75	1120.56	499.29	47	855.01	322.38	0.001^*^	33	908.85	300.38	33	878.81	353.47	0.711
HAMA	75	19.16	9.48	47	17.37	10.57	0.129	33	17.39	8.69	33	15.88	9.11	0.492
HAMD	75	17.45	7.46	47	16.48	8.71	0.518	33	15.91	7.01	33	16.15	8.52	0.900
Anxiety/somatization	75	3.54	2.65	47	2.98	2.55	0.224^a^	33	3.67	2.52	33	3.33	2.44	0.599^a^
Weight loss	75	0.18	0.49	47	0.22	0.56	0.733^a^	33	0.15	0.44	33	0.18	0.53	0.964^a^
Cognitive disturbances	75	2.76	2.55	47	2.91	2.78	0.876^a^	33	2.27	1.70	33	3.21	2.74	0.159^a^
Circadian fluctuations	75	0.69	0.80	47	0.56	0.72	0.427^a^	33	0.18	0.39	33	0.09	0.29	0.286^a^
Retardation symptoms	75	2.49	1.93	47	2.09	1.78	0.260^a^	33	2.18	1.57	33	2.24	1.82	0.927^a^
Sleep disturbances	75	3.42	2.13	47	2.98	2.26	0.313^a^	33	3.78	1.88	33	3.09	2.23	0.213^a^
Hopelessness symptoms	75	3.94	2.66	47	3.78	3.07	0.548^a^	33	3.12	1.90	33	3.33	2.12	0.826^a^
Somatic anxiety	75	9.20	6.03	47	7.26	5.17	0.072^a^	33	8.55	5.28	33	6.91	4.49	0.192^a^
Psychic anxiety	75	10.20	5.56	47	8.15	6.03	0.033^a^	33	8.85	4.87	33	8.87	5.72	0.777^a^

LEDD, levodopa equivalent daily dose; MDS-UPDRS-III, Movement Disorders Society-Unified Parkinson's Disease Rating Scale; HAMA, Hamilton Rating Scale for Anxiety; HAMD, Hamilton Rating Scale for Depression. ^*a*^Mann-Whitney U-tests, with FDR correction for multiple comparisons using for HAMA and HAMD domines.

Based on propensity score matching, a sub-cohort of 66 patients was obtained, including 33 patients for each treatment group in a 1:1 ratio (Table 1). Diagnostic statistics indicated a good balance of all demographic and main clinical baseline parameters between the reducing LEDD and non-reducing LEDD groups of the matched cohort. Accordingly, no significant difference was found for these parameters between the two groups. Furthermore, no significant difference was observed between the groups regarding HAMA and HAMD domain scores. The programming parameters are balanced in the two groups (Supplementary Table 2).

### Differences of outcomes at follow-up

Both groups showed improvement in HAMD total scores and HAMD sleep disturbance scores improved, but more improvement was found in the non-reducing group. Compared to the group with reduced LEDD, the group without LEDD reduction exhibited a significant decrease in the total HAMD score (*P* = 0.037). Additionally, the overall improvement rate of the HAMD score was significantly higher in the group without LEDD reduction when compared to the LEDD reduction group (*P* = 0.030).

The sleep disturbances domain of HAMD was significantly lower at the short-term follow-up in the non-reducing LEDD group (*P* = 0.009, *post-hoc*). As expected, the LEDDs were significantly lower (Table 2). Programming parameters are listed in Table 3 and were balanced in both groups. Long-term follow-up is shown in Supplementary Table 1. The patient's anxiety (*P* = 0.011) and depression (*P* = 0.013) improved significantly, but there is no different significantly between the two groups.

**Table 2:** Outcomes at baseline and at 3–6 months follow-up in unreducing and reducing LEDD groups for the matched cohort.

	**Unreducing LEDD**						**Reducing LEDD**						**Groups**
	**Baseline**			**Follow-up**		*P* ^a^	**Baseline**			**Follow-up**		*P* ^a^	*P* ^b^
	***n***	**Mean**	**SD**	**Mean**	**SD**		***n***	**Mean**	**SD**	**Mean**	**SD**		
MDS-UPDRS-III (med on)	33	23.01	10.94	18.97	14.24	0.026^*^	33	23.96	16.15	16.27	10.42	0.005^*^	0.469
MDS-UPDRS-III (med off)	33	54.26	24.17	31.86	20.00	< 0.001^*^	33	53.54	18.04	30.22	14.26	< 0.001^*^	0.898
Drug improvement	33	0.564	0.156	0.42	0.18	0.004^*^	33	0.577	0.206	0.44	0.33	0.027^*^	0.182
DBS improvement	33			0.40	0.27	-	33	-	-	0.41	0.29	-	0.912
DBS and improvement	33			0.65	0.15	-	33			0.69	0.18	-	0.246
LEDD	33	878.81	353.47	956.47	368.21	0.001^*^	33	908.85	300.38	655.49	248.99	< 0.001^*^	< 0.001^*^
HAMA	32	16.38	8.79	11.15	7.05	0.004^*^	33	17.39	8.69	14.03	7.30	0.070	0.148
HAMA improvement	32			0.18	0.48	-	33			0.07	0.58	-	0.508
HAMD	33	15.91	8.37	9.85	5.59	< 0.001^*^	33	16.39	6.78	13.33	7.57	0.067	0.037^*^
HAMD improvement	33			0.36	0.34	-	33			−0.01	0.74	-	0.030^*^
Anxiety/somatization	33	3.33	2.45	2.36	1.64	0.018^*^	33	3.67	2.52	3.15	2.51	0.437	0.315^b^
Weight loss	33	0.18	0.53	0.03	0.17	0.063	33	0.15	0.44	0.09	0.38	0.687	0.575^b^
Cognitive disturbances	33	3.21	2.78	1.52	1.37	0.014^*^	33	2.27	1.70	1.76	1.97	0.207	0.662^b^
Circadian fluctuation	33	0.12	0.33	0.61	0.75	0.014^*^	33	0.18	0.39	0.42	0.66	0.228	0.488^b^
Retardation symptoms	33	2.27	1.92	1.55	1.48	0.063	33	2.18	1.57	2.30	1.63	0.835	0.194^b^
Sleep disturbances	33	3.09	2.23	1.82	1.59	0.018^*^	33	3.79	1.88	3.15	2.18	0.228	0.043^*b^
Hopelessness symptoms	33	3.33	2.12	2.30	1.96	0.018^*^	33	3.12	1.90	2.45	1.75	0.207	0.744^b^
Somatic anxiety	33	8.55	5.28	6.67	4.38	0.042^*^	33	6.91	4.49	4.75	3.95	0.125	0.110^b^
Psychic anxiety	33	8.85	4.87	7.36	4.17	0.042^*^	33	8.87	5.72	6.39	4.42	0.125	0.363

LEDD, levodopa equivalent daily dose; MDS-UPDRS-III, Movement Disorders Society-Unified Parkinson's Disease Rating Scale; HAMA, Hamilton Rating Scale for Anxiety; HAMD, Hamilton Rating Scale for Depression. Drug improvement, [(med off–med on)/med off]; DBS improvement, [(baseline–follow-up)/baseline]. ^a^Wilcoxon signed-rank test, respectively, paired t-test, when parametric tests were applicable, with FDR correction for multiple comparisons using for HAMA and HAMD domines. ^b^Mann-Whitney U test, respectively, unpaired t-test, when parametric tests were applicable, with raw p-values. FDR correction for multiple comparisons used for HAMA and HAMD domines. ^*^P < 0.05 or *P* was significant after FDR correction.

**Table 3:** Effect size (CI) and relative changes for the matched cohort.

	**Effect size (CI)**		**Classification**		**Relative changes**	
	**Reducing LEDD**	**Unreducing LEDD**	**Reducing LEDD**	**Unreducing LEDD**	**Reducing LEDD**	**Unreducing LEDD**
MDS-UPDRS_III_med_on	0.57 (0.20 to 0.93)	0.28 (−0.67 to 0.63)	Moderate	Small	−0.32	−0.18
MDS-UPDRS_III_med_off	1.46 (0.96 to 1.94)	1.43 (0.94 to 1.91)	Large	Large	−0.44	−0.41
LEDD	1.19 (0.74 to 1.64)	−0.66 (−1.03 to −0.28)	Large	-	−0.28	0.09
HAMA	0.33 (−0.03 to 0.67)	0.49 (0.13 to 0.85)	Small	Small	−0.19	−0.30
HAMD	0.28 (−0.07 to 0.63)	0.72 (0.33 to 1.10)	Small	Moderate	−0.19	−0.38
Somatic anxiety	0.27 (−0.08 to 0.62)	0.42 (0.06 to 0.77)	Small	Small		
Psychic anxiety	0.30 (−0.05 to 0.65)	0.41 (0.05 to 0.76)	Small	Small		
Anxiety/somatization	0.16 (−0.18 to 0.50)	0.53 (0.16 to 0.89)	-	Moderate	−0.14	−0.29
Weight loss	0.05 (−0.29 to 0.39)	0.34 (−0.11 to 0.69)	-	Small	−0.40	−0.83
Cognitive disturbances	0.22 (−0.13 to 0.57)	0.62 (0.25 to 0.99)	Small	Moderate	−0.23	−0.54
Circadian fluctuation	0.32 (−0.03 to 0.67)	0.03 (−0.31 to 0.38)	Small	-	1.33	4
Retardation symptoms	−0.07 (−0.41 to 0.27)	0.33 (−0.02 to 0.68)	-	Small	0.06	−0.32
Sleep disturbances	0.28 (−0.07 to 0.62)	0.55 (0.18 to 0.91)	Small	Moderate	−0.17	−0.41
Hopelessness symptoms	0.39 (0.03 to 0.74)	0.50 (0.14 to 0.86)	Small	Moderate	−0.21	−0.31

CI, confidence interval; LEDD, levodopa equivalent daily dose; MDS-UPDRS-III, Movement Disorders Society-Unified Parkinson's Disease Rating Scale; HAMA, Hamilton Rating Scale for Anxiety; HAMD, Hamilton Rating Scale for Depression. Effect size, (mean Test_baseline_-mean Test_follow − up_)/SD Test_changescore_. ES: “small” (0.20–0.49), “moderate” (0.50–0.79), and “large” (≥0.80). Relative changes, [(mean Test_follow − up_-mean Test_baseline_)/mean Test_baseline_]. To evaluate the strength of clinical responses, effect size [(mean Test_baseline_-mean Test_follow − up_)/SD Test_changescores_] were calculated. Confidence intervals of effect sizes were calculated based on non-central t distribution.

UPDRS-III (MedOFF) and UPDRS-III (MedON) significantly improved from baseline to follow-up in the non-reducing LEDD (*P* < 0.001, *P* = 0.026; Table 2) and reducing LEDD groups (*P* < 0.001, *P* = 0.005; see Table 2). LEDD was significantly reduced by ~28% in the reducing LEDD group (*P* < 0.001). In addition, the drug improvement rate was significantly decreased in the two groups (non-reducing LEDD, *P* = 0.004; reducing LEDD, *P* = 0.027). HAMA and HAMD scores were significantly improved in the non-reducing LEDD group (HAMA, *P* = 0.004; HAMD, *P* < 0.001). No significant difference was found in the reducing LEDD group dealing with anxiety and depression (HAMA, *P* = 0.070; HAMD, *P* = 0.067).

**Figure 2:**
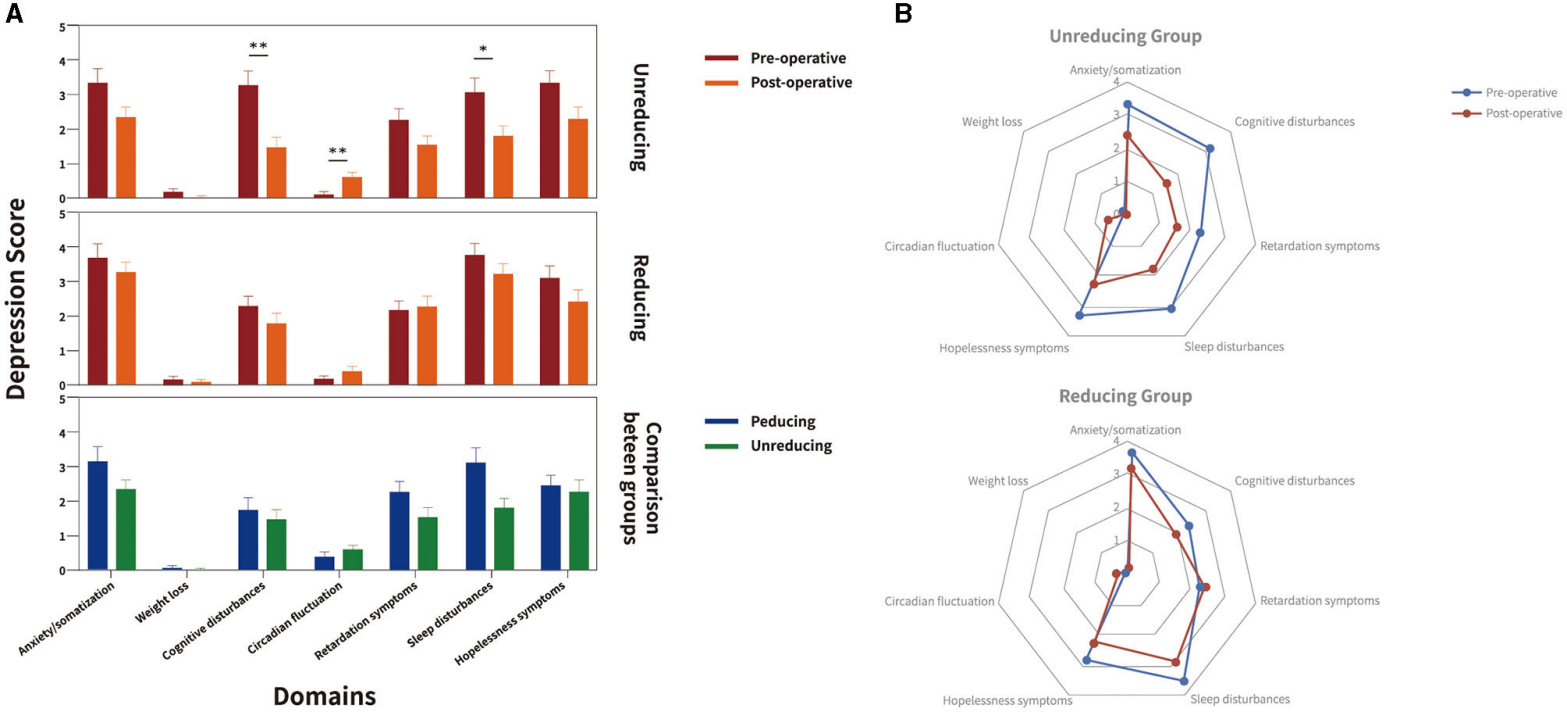
Depression domains at baseline and follow-up in the matched cohort for unreducing and reducing LEDD group. Figure illustrates Hamilton Rating Scale for Depression (HAMD) domains at baseline (red) and follow-up (orange) from baseline to follow-up, and the reducing (blue) and unreducing (green) groups in comparison between groups in **(A)** clustered box-plots and **(B)** radar charts. Significant intragroup improvements of HAMD domains from baseline to follow-up and between reducing and unreducing group are highlighted with black stars. In unreducing LEDD group, significant beneficial effects were observed for cognitive disturbances, sleep disturbances, the circadian fluctuation was significantly worse than before. In the reducing LEDD group, no significant change was observed between baseline and follow-up. The sleep disturbances domain of HAMD was significantly lower in unreducing LEDD group. *P < 0.05.

*Post-hoc* analyses of HAMD domain scores revealed differential effects for the two treatment groups (Figure 2): In the non-reducing LEDD group, significant beneficial effects were observed for cognitive disturbances (*P* = 0.003) and sleep disturbances (*P* = 0.012), but the circadian fluctuation was significantly worse than before (*P* = 0.004). In the reducing LEDD group, we found no significant change between baseline and follow-up.

Effect sizes (CI) were large for MDS-UPDRS-III (MedOFF) and small for HAMA in both groups. In the non-reducing LEDD group, confidence intervals (CIs) were small for MDS-UPDRS-III (MedON) and moderate for HAMD. In the reducing LEDD group, effect sizes were moderate for MDS-UPDRS-III (MedON) and small for HAMD. In the non-reducing LEDD group, all effect sizes of depression domains were larger. Comparing relative changes of the two groups, we observed more beneficial effects of the non-reducing LEDD group regarding the total scores of depressions and anxiety, domains of depression including anxiety/somatization, weight loss, cognitive disturbances, retardation symptoms, sleep disturbances, and hopelessness symptoms (Table 3). The adjustment of various classes of drugs in the drug-reducing group is shown in Table 4.

**Table 4:** The differences of anti-PD drugs.

	**Pre**		**Post**		**Reduction (%)**		***P***	
	**Reducing LEDD**	**Unreducing LEDD**	**Reducing LEDD**	**Unreducing LEDD**	**Reducing LEDD**	**Unreducing LEDD**	**Reducing LEDD**	**Unreducing LEDD**
LEDD	908.85 ± 300.38	878.81 ± 353.47	655.49 ± 248.99	956.47 ± 368.21	26.8 ± 19.07	−9.83 ± 15.63	< 0.001	0.001
Levodopa (LD)	761.48 ± 229.04	648.61 ± 214.62	529.03 ± 185.69	667.80 ± 259.17	29.18 ± 19.05	−8.10 ± 15.11	< 0.001^*^	0.583
Dopamine-agonists (DA)	67.05 ± 72.74	82.20 ± 79.67	51.55 ± 54.35	86.74 ± 81.40	16.13 ± 41.86	0.51 ± 21.84	0.153^a^	0.279^a^
Catechol-O-methyl transferase inhibitor (COMT-I)	43.29 ± 109.65	93.39 ± 166.01	25.06 ± 89.49	86.56 ± 155.11	7.07 ± 24.66	1.52 ± 8.70	0.109^a^	0.317^a^
Monoamine oxidase B inhibitor (MAOB-I)	9.09 ± 29.19	7.58 ± 25.38	3.03 ± 12.12	7.58 ± 25.38	6.06 ± 24.23	0.00 ± 0.00	0.180^a^	1.000^a^
Amantadine	53.03 ± 95.15	40.91 ± 82.40	54.55 ± 89.59	42.42 ± 83.97	7.83 ± 24.82	−1.52 ± 8.70	0.891^a^	0.317^a^

^a^Wilcoxon signed-rank test, respectively, paired t-test, when parametric tests were applicable. ^*^P < 0.05 after FDR correction.

## Discussion

The current longitudinal cohort study shows that: (1) bilateral STN-DBS has significant beneficial effects on global and specific aspects of neuropsychiatric symptoms and UPDRS-III at short-term follow-up; (2) the short-term effects of DBS on neuropsychiatric symptoms are closely related to LEDD adjustment.

This is the first report of neuropsychiatric symptoms in PD patients during short-term follow-ups. We supplemented the existing medication adjustment model and found that maintaining early medication stability is more favorable for improving patients' psychiatric symptoms. PSM could balance the differences between the two groups and achieve RCT-like results. It represents the effects of these treatments as seen in clinical practice, and as such, we believe the data has real clinical significance.

STN-DBS improved motor symptoms, which was consistent with previous research (41). At the 1-year follow-up, STN-DBS improved neuropsychiatric symptoms (Supplementary Table 1). STN-DBS improves depression in PD patients in a multifactorial way that is related to motor function, anti-PD drugs, and contact location. First and foremost, improvement in motor symptoms promotes emotional relief (42). Furthermore, STN-DBS may influence monoaminergic structures (serotonergic raphe nucleus and noradrenergic locus coeruleus) to reduce depression (43, 44). Furthermore, disruption of DA transmission is important in PD patients with neuropsychiatric symptoms. Stimulation after STN-DBS may gradually restore the patient's DA interruption by increasing GABAergic transmission (45). However, neural remodeling may necessitate long-term stimulation to achieve therapeutic effects, which is consistent with our findings of long-term improvement in anxiety and depression. Finally, the anterior, medial, and ventral STN were more conducive to alleviating patients' neuropsychiatric symptoms (46). Stimulating these areas can have a preferential effect on the limbic pathways, improving the patient's depression symptoms. These systems are involved in the regulation of neuropsychiatric symptoms and may explain why long-term depression is improving.

STN-DBS can reduce anxiety and depression in the short term (47). Non-reducing LEDD outperformed reducing LEDD when relative changes and effect sizes were considered. The non-reducing LEDD group was more conducive to improving patients' neuropsychiatric symptoms in the short term, which could be due to a variety of factors. Depression is caused by depleted levels of serotonin, dopamine, and norepinephrine (NE). Furthermore, the hypothalamic-pituitary adrenal axis and hypothalamic-pituitary thyroid axis both contribute to depression pathophysiology (48, 49). Among the several types of drugs used to treat PD patients, MAO inhibitors can inhibit neurotransmitter reuptake [5-HT, NE, and dopamine (DA)], and levodopa can directly increase the patient's dopaminergic levels (50, 51). Therefore, dopaminergic treatment can alleviate anxiety and depression. However, COMT-I and amantadine may exacerbate depressive symptoms (52). According to our findings, an early medication reduction was primarily focused on the reduction of levodopa. The primary target of early postoperative drug reduction is not COMT-I and amantadine (Table 4). As a result, early drug discontinuation is not beneficial to the improvement of neuropsychiatric symptoms. This finding provides a rationale for the timing of drug reduction after DBS and opposes premature drug reduction after DBS.

Finally, there was no significant change in anxiety and depression symptoms in the early drug reduction group in the short-term postoperative period. This group's mood was not affected by the medication reduction. This finding could be attributed to the improvement of the patient's motor symptoms following DBS, which can reduce psychological distress and improve clinical symptoms, and the improvement of motor disorders allows for mindfulness-based interventions, which can reduce depression (53).

Although existing drug reduction strategies are primarily based on programmed efficacy, there was no significant difference in the programming parameters between the two groups of patients (Supplementary Table 2). Patients with mid-to-advanced PD who receive STN-DBS are frequently bothered by financial issues and levodopa-induced dyskinesia caused by high-dose, multi-type drug therapy (54, 55). Early programming is made more difficult by patients' urgent need to reduce drug dosage to improve quality of life (18, 56, 57). Most programming doctors struggle with how to quickly find the balance between optimal stimulation parameters and drug adjustment timing in a massive combination of a large number of programmed parameters and drug adjustments (10, 55). Our study has revealed that a substantial reduction in medication during the initial programming can impede the psychiatric outcomes of STN-DBS. To mitigate this scenario, it is essential to conduct multiple programming sessions and medication adjustments over the course of several weeks, gradually working toward optimizing result.

### Limitations

This retrospective study primarily elucidates the phenomenon that maintaining early medication stability yields greater clinical benefits. However, it does not provide a definitive optimal timeframe for medication reduction. The practice of reducing medication only at two time points, during the initial programming and 3–6 months later, is evidently suboptimal. A more physiologically approach to postoperative programming and medication adjustment would involve gradual changes. Unfortunately, due to a large number of patients and geographical reasons related to patient residence, such an approach was not feasible in our study. In the future, remote programming may offer a solution to this issue.

## Conclusions

In conclusion, (1) It was discovered that maintaining drug stability within 3–6 months is more conducive to neuropsychiatric symptom improvement; (2) Providing evidence for drug reduction timing and opposing early drug reduction after surgery; (3) one step and probably a major drug reduction at the first post-op programming will hamper the mental outcome in STN-DBS. To avoid this, the process needs fine tune individually.

## Data availability statement

The original contributions presented in the study are included in the article/Supplementary material, further inquiries can be directed to the corresponding authors.

## Ethics statement

The studies involving humans were approved by Beijing Tiantan Ethics Committee. The studies were conducted in accordance with the local legislation and institutional requirements. The participants provided their written informed consent to participate in this study.

## Author contributions

YD: Data curation, Formal analysis, Methodology, Software, Writing—original draft. TH: Writing—review and editing, Data curation. HX: Data curation, Writing—review and editing. HF: Data curation, Writing—review and editing. FM: Project administration, Writing—review and editing. AY: Methodology, Visualization, Writing—review and editing. YB: Data curation, Formal analysis, Investigation, Software, Visualization, Writing—review and editing. JZ: Conceptualization, Funding acquisition, Project administration, Resources, Writing—review and editing.
