# Supplementary material for: Premature drug reduction after subthalamic nucleus deep brain stimulation leading to worse depression in patients with Parkinson's disease
Source: Front Neurol. 2023 Oct 19;14:1270746. doi: 10.3389/fneur.2023.1270746 (PMC10620523; doi:10.3389/fneur.2023.1270746)
Supplement: Supplementary file 1 [file Table_1.DOCX]

Supplementary materials

Supplementary Table 1: Outcomes at 1-year follow-up in all patients for the matched cohort.

|  | Pre | 1 year follow-up | P |
| --- | --- | --- | --- |
| Anxiety | 16.32±9.60 | 11.48±6.94 | 0.011^*^ |
| Depression | 15.06±6.71 | 12.39±1.52 | 0.013^*^ |

^b^ Wilcoxon signed-rank test.

^*^ P<0.05

Supplementary Table2: programming parameters

|  | Pulse width (left) | Voltage (left) | Frequency (left) | Pulse width (right) | Voltage (right) | Frequency (right) |
| --- | --- | --- | --- | --- | --- | --- |
| Reducing LEDD | 62.50±4.42 | 1.71 ±0.30 | 130.00 ± 12.07 | 59.13 ± 11.25 | 1.70 ± 0.34 | 130.00 ± 12.34 |
| Unreducing LEDD | 60.38 ±1.96 | 1.70 ± 0.33 | 129.04 ± 14.90 | 60.77 ±2.72 | 1.71 ± 0.27 | 129.04 ±14.9 |

Supplementary Table3: anti-PD drugs for each patient

| Pre DBS | | | | | 3-6 months follow-up | | | | | group |
| --- | --- | --- | --- | --- | --- | --- | --- | --- | --- | --- |
| levodopa(LD) | COMT-I | MAO-BI | DA | amantadine | levodopa(LD) | COMT-I | MAO-BI | DA | amantadine |  |
| 1013.91 | 0.00 | 0 | 75 | 0 | 732.00 | 0.00 | 0 | 0 | 0 | Reducing LEDD |
| 775.94 | 0.00 | 0 | 0 | 0 | 681.95 | 0.00 | 0 | 0 | 0 | Reducing LEDD |
| 487.97 | 225.56 | 0 | 112.5 | 0 | 300.00 | 225.56 | 0 | 112.5 | 0 | Reducing LEDD |
| 787.97 | 0.00 | 0 | 100 | 0 | 689.97 | 0.00 | 0 | 100 | 0 | Reducing LEDD |
| 787.97 | 0.00 | 0 | 25 | 0 | 693.98 | 0.00 | 0 | 50 | 0 | Reducing LEDD |
| 751.88 | 0.00 | 50 | 75 | 200 | 563.91 | 0.00 | 50 | 75 | 200 | Reducing LEDD |
| 775.94 | 0.00 | 0 | 0 | 200 | 387.97 | 0.00 | 0 | 0 | 200 | Reducing LEDD |
| 387.97 | 0.00 | 0 | 0 | 0 | 200.00 | 0.00 | 0 | 0 | 0 | Reducing LEDD |
| 751.88 | 0.00 | 50 | 100 | 0 | 563.91 | 0.00 | 50 | 100 | 0 | Reducing LEDD |
| 637.97 | 0.00 | 150 | 150 | 0 | 487.97 | 0.00 | 0 | 150 | 0 | Reducing LEDD |
| 787.97 | 0.00 | 0 | 25 | 0 | 781.95 | 0.00 | 0 | 12.5 | 0 | Reducing LEDD |
| 751.88 | 0.00 | 0 | 200 | 300 | 751.88 | 0.00 | 0 | 50 | 200 | Reducing LEDD |
| 975.94 | 0.00 | 0 | 0 | 0 | 693.98 | 0.00 | 0 | 0 | 0 | Reducing LEDD |
| 567.97 | 0.00 | 0 | 100 | 0 | 550.00 | 0.00 | 0 | 0 | 100 | Reducing LEDD |
| 1153.35 | 451.13 | 0 | 75 | 150 | 543.98 | 451.13 | 0 | 75 | 150 | Reducing LEDD |
| 881.95 | 0.00 | 0 | 75 | 100 | 850.75 | 0.00 | 0 | 75 | 0 | Reducing LEDD |
| 743.98 | 225.56 | 0 | 0 | 200 | 300.00 | 0.00 | 0 | 150 | 150 | Reducing LEDD |
| 381.95 | 0.00 | 0 | 0 | 0 | 287.97 | 0.00 | 0 | 0 | 0 | Reducing LEDD |
| 986.47 | 0.00 | 0 | 150 | 0 | 800.00 | 0.00 | 0 | 150 | 0 | Reducing LEDD |
| 493.98 | 0.00 | 0 | 150 | 0 | 300.00 | 0.00 | 0 | 150 | 0 | Reducing LEDD |
| 387.97 | 0.00 | 0 | 0 | 0 | 300.00 | 0.00 | 0 | 0 | 0 | Reducing LEDD |
| 563.91 | 0.00 | 0 | 300 | 0 | 300.00 | 0.00 | 0 | 75 | 0 | Reducing LEDD |
| 863.91 | 0.00 | 0 | 0 | 0 | 300.00 | 0.00 | 0 | 0 | 200 | Reducing LEDD |
| 563.91 | 0.00 | 0 | 75 | 0 | 563.91 | 0.00 | 0 | 75 | 0 | Reducing LEDD |
| 1081.95 | 0.00 | 0 | 75 | 0 | 480.00 | 0.00 | 0 | 75 | 0 | Reducing LEDD |
| 882.71 | 300.75 | 0 | 100 | 100 | 750.00 | 0.00 | 0 | 100 | 100 | Reducing LEDD |
| 687.97 | 0.00 | 0 | 0 | 0 | 437.97 | 0.00 | 0 | 0 | 0 | Reducing LEDD |
| 637.97 | 0.00 | 0 | 0 | 0 | 437.97 | 0.00 | 0 | 0 | 0 | Reducing LEDD |
| 1363.91 | 0.00 | 0 | 0 | 0 | 450.00 | 0.00 | 0 | 0 | 0 | Reducing LEDD |
| 563.91 | 0.00 | 50 | 100 | 0 | 400.00 | 0.00 | 0 | 1 | 0 | Reducing LEDD |
| 975.94 | 225.56 | 0 | 0 | 0 | 781.95 | 150.38 | 0 | 0 | 0 | Reducing LEDD |
| 975.94 | 0.00 | 0 | 150 | 200 | 600.00 | 0.00 | 0 | 100 | 200 | Reducing LEDD |
| 693.98 | 0.00 | 0 | 0 | 300 | 493.98 | 0.00 | 0 | 25 | 300 | Reducing LEDD |
| 787.97 | 451.13 | 0 | 112.5 | 200 | 937.97 | 451.13 | 0 | 112.5 | 200 | Unreducing LEDD |
| 281.95 | 0.00 | 0 | 0 | 0 | 281.95 | 0.00 | 0 | 0 | 0 | Unreducing LEDD |
| 375.94 | 150.38 | 0 | 100 | 200 | 375.94 | 150.38 | 0 | 100 | 200 | Unreducing LEDD |
| 693.98 | 0.00 | 0 | 150 | 0 | 693.98 | 0.00 | 0 | 150 | 0 | Unreducing LEDD |
| 1137.97 | 225.56 | 0 | 175 | 0 | 1337.97 | 225.56 | 0 | 175 | 0 | Unreducing LEDD |
| 775.94 | 0.00 | 0 | 0 | 0 | 775.94 | 0.00 | 0 | 0 | 0 | Unreducing LEDD |
| 751.88 | 0.00 | 0 | 300 | 0 | 1127.82 | 0.00 | 0 | 250 | 0 | Unreducing LEDD |
| 481.95 | 0.00 | 0 | 0 | 0 | 481.95 | 0.00 | 0 | 0 | 0 | Unreducing LEDD |
| 479.95 | 225.56 | 0 | 0 | 0 | 694.36 | 225.56 | 0 | 0 | 0 | Unreducing LEDD |
| 1051.88 | 0.00 | 0 | 0 | 0 | 1051.88 | 0.00 | 0 | 0 | 0 | Unreducing LEDD |
| 831.95 | 0.00 | 0 | 0 | 0 | 831.95 | 0.00 | 0 | 0 | 0 | Unreducing LEDD |
| 475.94 | 0.00 | 0 | 150 | 0 | 475.94 | 0.00 | 0 | 150 | 0 | Unreducing LEDD |
| 774.06 | 300.00 | 0 | 187.5 | 0 | 774.06 | 300.00 | 0 | 187.5 | 0 | Unreducing LEDD |
| 581.95 | 0.00 | 0 | 0 | 100 | 581.95 | 0.00 | 0 | 0 | 150 | Unreducing LEDD |
| 975.94 | 0.00 | 0 | 150 | 200 | 1163.91 | 0.00 | 0 | 150 | 200 | Unreducing LEDD |
| 587.97 | 0.00 | 0 | 100 | 0 | 587.97 | 0.00 | 0 | 150 | 0 | Unreducing LEDD |
| 587.97 | 0.00 | 0 | 75 | 0 | 587.97 | 0.00 | 0 | 75 | 0 | Unreducing LEDD |
| 563.91 | 451.13 | 0 | 0 | 0 | 751.88 | 451.13 | 0 | 0 | 0 | Unreducing LEDD |
| 475.94 | 0.00 | 0 | 150 | 0 | 475.95 | 0.00 | 0 | 150 | 0 | Unreducing LEDD |
| 487.97 | 0.00 | 100 | 0 | 0 | 487.97 | 0.00 | 100 | 75 | 0 | Unreducing LEDD |
| 875.94 | 0.00 | 0 | 75 | 0 | 1125.94 | 0.00 | 0 | 75 | 0 | Unreducing LEDD |
| 751.88 | 451.13 | 0 | 200 | 0 | 1016.64 | 225.56 | 0 | 200 | 0 | Unreducing LEDD |
| 637.97 | 0.00 | 0 | 37.5 | 0 | 637.97 | 0.00 | 0 | 37.5 | 0 | Unreducing LEDD |
| 781.95 | 451.13 | 0 | 150 | 200 | 781.95 | 451.13 | 0 | 150 | 200 | Unreducing LEDD |
| 487.97 | 0.00 | 0 | 75 | 0 | 581.95 | 0.00 | 0 | 75 | 0 | Unreducing LEDD |
| 287.97 | 375.94 | 100 | 0 | 0 | 587.97 | 375.94 | 100 | 0 | 0 | Unreducing LEDD |
| 581.95 | 0.00 | 50 | 200 | 200 | 581.95 | 0.00 | 50 | 300 | 200 | Unreducing LEDD |
| 575.94 | 0.00 | 0 | 0 | 0 | 575.94 | 0.00 | 0 | 0 | 0 | Unreducing LEDD |
| 581.95 | 0.00 | 0 | 75 | 0 | 581.95 | 0.00 | 0 | 75 | 0 | Unreducing LEDD |
| 393.98 | 0.00 | 0 | 0 | 0 | 393.98 | 0.00 | 0 | 0 | 0 | Unreducing LEDD |
| 869.92 | 0.00 | 0 | 75 | 250 | 869.92 | 0.00 | 0 | 75 | 250 | Unreducing LEDD |
| 937.97 | 0.00 | 0 | 75 | 0 | 787.97 | 0.00 | 0 | 75 | 0 | Unreducing LEDD |
| 375.94 | 0.00 | 0 | 100 | 0 | 400.00 | 0.00 | 0 | 100 | 0 | Unreducing LEDD |

Supplementary Table4: The Pearson correlation analysis between DA, COMT-I, and MAOB-I and HAMA and HAMD

|  | COMT-I | MAOB-I | DA |
| --- | --- | --- | --- |
| Pre HAMA in Reducing LEDD | R = -0.053 P = 0.770 | R = -0.076 P = 0.674 | R = 0.069 P = 0.701 |
| Pre HAMD in Reducing LEDD | R = -0.283 P = 0.111 | R = -0.224 P = 0.210 | R = -0.096 P = 0.597 |
| Follow-up HAMA in Reducing LEDD | R = 0.017 P = 0.926 | R = -0.142 P = 0.429 | R = -0.146 P = 0.418 |
| Follow-up HAMD in Reducing LEDD | R = -0.054 P = 0.764 | R = -0.250 P = 0.161 | R = -0.224 P = 0.210 |
| Pre HAMA in Unreducing LEDD | R = -0.033 P = 0.857 | R = -0.090 P = 0.616 | R = -0.272 P = 0.126 |
| Pre HAMD in Unreducing LEDD | R = -0.053 P = 0.771 | R = -0.136 P = 0.449 | R = -0.343 P = 0.050 |
| Follow-up HAMA in Unreducing LEDD | R = 0.027 P = 0.883 | R = -0.119 P = 0.518 | R = -0.040 P = 0.829 |
| Follow-up HAMD in Unreducing LEDD | R = -0.051 P = 0.778 | R = -0.080 P = 0.659 | R = -0.328 P = 0.063 |
