# Supplementary material for: Premature drug reduction after subthalamic nucleus deep brain stimulation leading to worse depression in patients with Parkinson's disease
Source: Front Neurol. 2023 Oct 19;14:1270746. doi: 10.3389/fneur.2023.1270746 (PMC10620523; doi:10.3389/fneur.2023.1270746)
Supplement: Supplementary file 2 [file Data_Sheet_1.ZIP › illustrate.docx]

Thank you for your request. As per your requirements, we have provided all the original data for the 122 enrolled patients and the detailed data for each of the 66 patients calculated after PSM matching. The dataset "122patients_cohort" contains the original data for all 122 enrolled patients. The data "0.03 - Anxiety subscore" and "0.03 - Depression subscore" represent the detailed original data of the 66 patients used for anxiety and depression calculations in FIGURE2. The data "0.03 - Screening scores" include control parameters, motion scores, and summary scores of various scales for all 66 matched patients. The data "0.03 - Medication usage details" summarize the different types of medication used by the 66 matched patients, as reported in table 4. Lastly, the data "0.03 - Long-term follow-up" consists of long-term follow-up data for all patients for over a year. As not all patients have complete long-term follow-up data, the results have been provided as supplementary information in the appendix.

Statistical analyses were conducted using SPSS statistical software for Windows, version 25.0 (SPSS, Chicago, IL, USA), and R 3.6.1 (R Foundation for Statistical Computing). Propensity score analysis was performed using the "MatchIt" package in R. The specific parameters for propensity score analysis have been thoroughly described in the methodology section, and you can use the same parameters in the R environment with the "MatchIt" package to replicate the analysis.
